# Enhanced energy density of PVDF-based nanocomposites via a core–shell strategy

**DOI:** 10.1038/s41598-020-73884-6

**Published:** 2020-10-13

**Authors:** JingJing Xu, Chao Fu, Huiying Chu, Xianyou Wu, Zhongyang Tan, Jing Qian, Weiyan Li, Zhongqian Song, Xianghai Ran, Wei Nie

**Affiliations:** 1 Lab of Polymer Composites Engineering, Changchun Institute of Applied Chemistry, Chinese Academy of Sciences, Changchun, 130022 China; 2 University of Science and Technology of China, Anhui, 230026 China

**Keywords:** Composites, Electronic properties and materials, Nanowires, Structural properties

## Abstract

In recent years, high energy density polymer capacitors have attracted a lot of scientific interest due to their potential applications in advanced power systems and electronic devices. Here, core–shell structured TiO_2_@SrTiO_3_@polydamine nanowires (TiO_2_@SrTiO_3_@PDA NWs) were synthesized via a combination of surface conversion reaction and in-situ polymerization method, and then incorporated into the poly(vinylidene fluoride) (PVDF) matrix. Our results showed that a small amount of TiO_2_@SrTiO_3_@PDA NWs can simultaneously enhance the breakdown strength and electric displacement of nanocomposite (NC) films, resulting in improved energy storage capability. The 5 wt% TiO_2_@SrTiO_3_@PDA NWs/PVDF NC demonstrates 1.72 times higher maximum discharge energy density compared to pristine PVDF (10.34 J/cm^3^ at 198 MV/m vs. 6.01 J/cm^3^ at 170 MV/m). In addition, the NC with 5 wt% TiO_2_@SrTiO_3_@PDA NWs also demonstrates an excellent charge–discharge efficiency (69% at 198 MV/m). Enhanced energy storage performance is due to hierarchical interfacial polarization among their multiple interfaces, the large aspect ratio as well as surface modification of the TiO_2_@SrTiO_3_ NWs. The results of this study provide guidelines and a foundation for the preparation of the polymer NCs with an outstanding discharge energy density.

## Introduction

Dielectric capacitors with the ultrafast charging and discharging speeds, high power density and low cost are very attractive materials for the potential applications in the pulsed power electronic devices, such as radars, lasers, rail guns, and medical defibrillators^1–7^. However, the dielectric capacitors have lower energy density than batteries, fuel cells, and double-layer supercapacitors so this type of energy storage device is still expensive and bulky^8–12^. For instance, the energy density of the biaxial-oriented polypropylenes (BOPP), the best commercially available dielectric material, is ~ 2 J/cm^3^, which is significantly lower than the energy density of a typical electrochemical capacitor (i.e. ~ 20 J/cm^3^). Therefore, to miniaturize and reduce the cost of high-power electronic devices, novel materials for dielectric capacitors with dramatically improved energy density are required. PVDF with highly electronegative fluorine atoms exhibits relatively high permittivity and might be a competent candidate to construct high energy density capacitors^13–15^.

The energy density (U) of a dielectric material is typically calculated using the following equation:^16^

1 $${\text{U}} = \smallint {\text{EdD }}$$

where E denotes the applied electrical field, and D is electric displacement, which can be calculated using the following equation for linear dielectrics:

2 $${\text{D}} = {\upvarepsilon }_{0} {\upvarepsilon }_{{\text{r}}} {\text{E}}$$

where ε_0_ is the permittivity of vacuum and ε_r_ is the relative permittivity of the materials. Thus, the breakdown strength and relative permittivity are important parameters to achieve high energy density. Among several available dielectric materials, ceramic/polymer nanocomposites (NCs) have attracted significant attention as they combine the advantages of ceramic fillers (high permittivity) and the polymer matrix (high breakdown strength, low dielectric loss, flexibility, and low cost)^11,17–19^. However, high content of ceramic particles, usually over 50 vol%, is needed to realize of a high enough permittivity in NCs, resulting in low breakdown strength.

Recent studies have shown that one-dimensional nanofillers with large aspect ratio, such as TiO_2_^6^, BaTiO_3_^20,21^, BaSrTiO_3_^22,23^, and SrTiO_3_^24^, are more effective than the nanoparticles counterparts in improving the permittivity and energy density of the dielectric NCs. One-dimensional nanofillers with a large aspect ratio can effectively alleviate the conflict between the raise of permittivity and the decline of breakdown strength. The main reason is that the smaller specific surface of one-dimensional nanofillers helps to reduce the surface energy, which prevents the agglomeration of nanofillers in the polymer matrix. Additionally, one-dimensional nanofillers act as ordered scattering centers for charges and increase the tortuosity of the breakdown path^25,26^.

TiO_2_ is convenient for large-scale preparation and has a moderate permittivity, which can reduce the permittivity contrast with polymer matrix when it is used as a filler. However, TiO_2_ possesses a high electrical conductivity, which increases the dielectric loss and reduces the energy efficiency of the TiO_2_/polymer NCs, especially at high TiO_2_ contents^3^. Paraelectric SrTiO_3_ ceramic material has high permittivity, low electrical conductivity and low remnant polarization, all of which can improve the energy storage capability of the NCs containing SrTiO_3_ as a filler^10,24^. In addition, the core–shell structured nanofillers can provide large electric displacement via additional polarization in the internal interfaces and might contribute to enhanced energy density of polymer matrix^3,25^. However, polymer nanocomposites, consisting of core–shell structured TiO_2_@SrTiO_3_ NWs as nanofillers, have seldom been reported.

In this work, PVDF is chosen as the polymer matrix because its permittivity is higher compared with that of other polymers^13–15,27^. We prepared novel core–shell TiO_2_@SrTiO_3_ NWs with the aim to combine the electrical properties of TiO_2_ and SrTiO_3_ and to obtain the NCs with high discharge energy density. Our NC design was based on the following assumptions and expectations: (1) Encapsulation of SrTiO_3_ outer shell inhibits the negative effects of TiO_2_ NWs on the NCs properties. (2) Paraelectric ceramic SrTiO_3_ decreases the remnant polarization of the NCs. (3) TiO_2_@SrTiO_3_ NWs would improve permittivity of the NCs better than bare TiO_2_ NWs, which can be ascribed to additional polarization of the internal interfaces of the nanofillers between crystallized TiO_2_ and SrTiO_3_. To better disperse TiO_2_@SrTiO_3_ NWs in the PVDF matrix and also to make it more compatible, dopamine was used as a surface modifier. Dielectric properties as well as energy storage capability and efficiency of TiO_2_@SrTiO_3_@PDA NWs/PVDF NCs were systematically studied. NC containing 5 wt% TiO_2_@SrTiO_3_@PDA NWs exhibits the highest discharge energy density value (i.e. 10.34 J/cm^3^) and maintains high charge–discharge efficiency (69% at 198 MV/m). Due to the addition of a small amount of the dopamine-modified TiO_2_@SrTiO_3_ NWs, the corresponding NCs show good mechanical properties. Due to their high energy storage capability, high energy efficiency and excellent mechanical properties, these NCs have the potential for future applications in advanced electric power systems and electronic devices.

## Material and experimental methods

### Materials

Kynar 301F PVDF with a density equal to 1.76 g/cm^3^ was purchased from Arkema. Molecular weight of PVDF is about 500,000. Strontium hydroxide octahydrate (Sr(OH)_2_·8H_2_O), anatase TiO_2_, Tris-(hydroxy-methyl)-aminomethane (Tris, 99%), dopamine hydrochloride (98%), N,N-dimethylformamide (DMF) and other reagents were provided by Aladdin (China).

### Synthesis of Na_2_Ti_3_O7 nanowires

The Na_2_Ti_3_O_7_ NWs were synthesized by a hydrothermal method as described elsewhere^28,29^. 5 g of anatase titanium dioxide nanopowder and 100 mL of 10 M sodium hydroxide aqueous solution were added into a beaker, sonicated for 10 min and then stirred vigorously at room temperature for 12 h. The mixture was then poured into a 150 mL teflon-autoclave and kept at 200 °C for 72 h. The obtained products were collected via centrifugation, dispersed and thoroughly washed with deionized water and ethanol for several times, respectively, followed by vacuum oven-drying at 80 °C for 12 h.

### Synthesis of TiO_2_ nanowires

The synthesis of TiO_2_ NWs was accomplished by using Na_2_Ti_3_O_7_ NWs as raw materials and following the literature procedure^30^. First, the synthesized Na_2_Ti_3_O_7_ NWs were dispersed in 500 mL of 0.2 M hydrochloric acid aqueous solution and soaked for 24 h. Afterward, the products were collected via centrifugation and dispersed and washed with deionized water and ethanol for several times, respectively, and then dried in a vacuum oven at 80 °C for 12 h. Finally, to obtain TiO_2_ NWs, the H_2_Ti_3_O_7_ NWs were heated for 3 h at 600 °C.

### Preparation of TiO_2_@SrTiO_3_ NWs

The TiO_2_@SrTiO_3_ NWs were synthesized by converting TiO_2_ NWs surface via hydrothermal method described in literature^31^. The synthesized TiO_2_ NWs were placed into a 150 mL teflon autoclave containing 100 mL of Sr(OH)_2_·8H_2_O aqueous solution. The autoclave was heated at 150 °C for 24 h. The obtained products were collected via centrifugation, dispersed and thoroughly washed with deionized water and ethanol for several times, respectively, followed by vacuum oven-drying at 80 °C for 12 h. The resulting products are denoted as TiO_2_@SrTiO_3_ NWs.

### Surface modification of nanowires

The TiO_2_@SrTiO_3_ NWs were added in 100 mL of 10 mM Tris-buffer solution (with pH = 8.5) and sonicated for 10 min. Afterward, 0.5 g of dopamine hydrochloride was added into the above suspension. The mixture was sonicated for another 10 min and stirred vigorously at 60 °C for 12 h. The resulting products were collected via centrifugation, dispersed and thoroughly washed with deionized water and ethanol for several times, respectively, followed by vacuum oven-drying at 80 °C overnight. The functionalized nanowires are denoted as TiO_2_@SrTiO_3_@PDA NWs.

### Preparation of nanocomposite films

**Figure 1:**
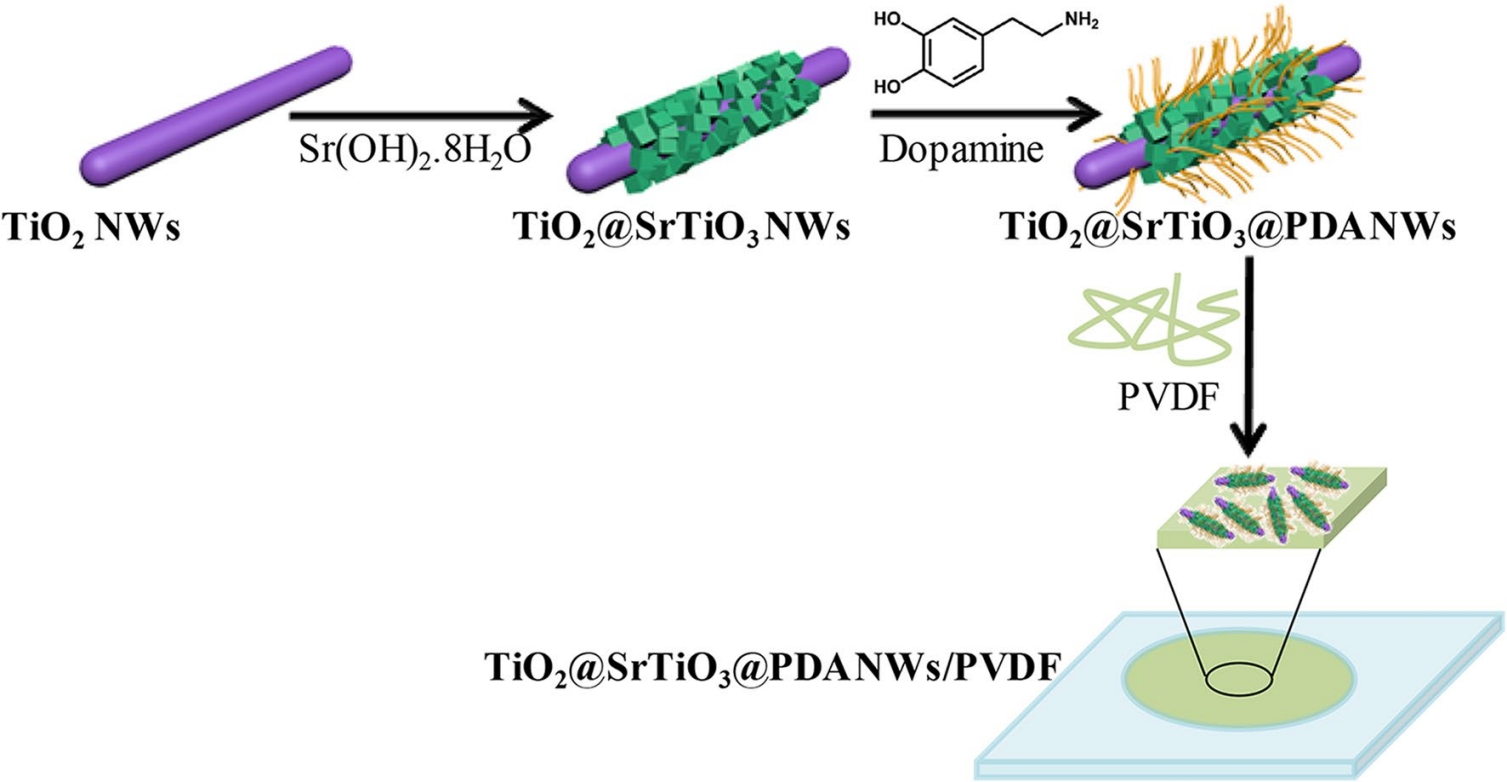
Fabrication scheme for TiO_2_@SrTiO_3_@PDA NWs/PVDF NCs. This figure was created using Autodesk 3D Studio Max 2014 (https://www.autodesk.com) and Microsoft Office PowerPoint 2007 (https://www.office.com).

In order to prepare NC films, first, PVDF was added into DMF and stirred vigorously at room temperature for 6 h to obtain a homogeneous solution. Then the given amount of TiO_2_@SrTiO_3_@PDA NWs was added in DMF and sonicated for 30 min, after which the PVDF solution was added to the above suspension. The mixture was stirred vigorously for 12 h, followed by sonication for 30 min, and then cast onto a smooth and clean glass substrate. The cast films were dried for 12 h in a vacuum at 60 °C to evaporate the residual solvent. NCs films with different contents of TiO_2_@SrTiO_3_@PDA NWs (1 wt%, 5 wt% and 15 wt%) were fabricated. For comparison, the 15 wt% SrTiO_3_@PDA NWs/PVDF NC and 15 wt% TiO_2_@PDA NWs/PVDF NC were also prepared using the same procedure. The NC films were about 50 μm thick. The procedure for fabrication of TiO_2_@SrTiO_3_@PDA NWs/PVDF NCs is demonstrated in Fig. 1.

### Characterization

Bruker Vertex 70 spectrometer was used to record the Fourier-transform infrared (FT-IR) spectra. XL30 scanning electron microscope (SEM) manufactured by FEI Co. (Netherlands) was used to analyze the morphology of the synthesized NWs and the NC film. The JEOL-1011 Transmission electron microscope (TEM) manufactured by JEOL Co. (Japan) was employed to analyze the morphology of the synthesized NWs. X-ray diffraction (XRD) was performed by the D8 Advanced diffractometer (Bruker, Germany) using CuKα radiation as an X-ray source with a 3°/min scanning rate. X-ray photoelectron spectroscopy (XPS) was performed using Thermo Scientific ESCALAB 250 to analyze the surface composition of the synthesized NWs. Thermogravimetric analysis (TGA) was done using Q500 analyzer (TA Co., USA) in the N_2_ atmosphere at a 10 °C/min heating rate. The crystallization behavior of the PVDF matrix was analyzed by differential scanning calorimetry (DSC) using the Q20 instrument (TA Co., USA) conducted in the N_2_ atmosphere in the 50–200 °C range at 10 °C/min heating and cooling rates.

The permittivity and loss of the NCs were obtained using Novocontrol Concept 40 broadband dielectric spectrometer. Measurements were performed at room temperature in the 100 Hz–1 MHz frequency range. Both sides of the samples were coated with silver paste to characterize the dielectric properties. The electric displacement−electric field (D–E) hysteresis measurements were conducted by the Precision Multiferroic Materials Analyzer manufactured by Radiant Co. (USA). Both sides of the samples were coated with gold, which acts as the electrodes for D–E hysteresis measurement. The diameter and thickness of the gold electrodes are 2 mm and 50–100 nm, respectively. The mechanical tensile properties were tested using a universal Instron 5869 machine (Instron Engineer Co., USA) at 1 mm/min strain rate.

## Results and discussion

### Characterization of the nanowires

**Figure 2:**
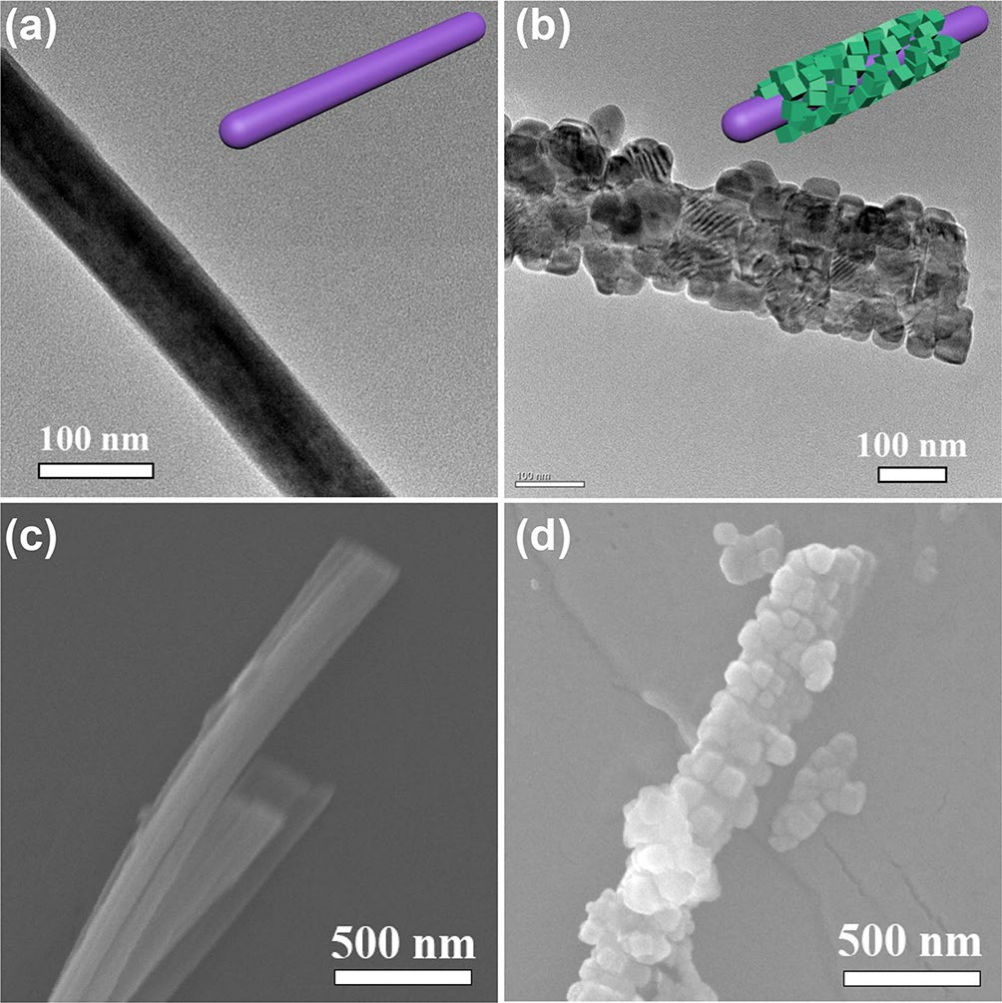
SEM images of (**a**) pristine TiO_2_ NWs and (**b**) TiO_2_@SrTiO_3_ NWs. TEM images of (**c**) pristine TiO_2_ NWs and (**d**) TiO_2_@SrTiO_3_ NWs. This figure was created using Autodesk 3D Studio Max 2014 (https://www.autodesk.com) and Microsoft Office PowerPoint 2007 (https://www.office.com).

**Figure 3:**
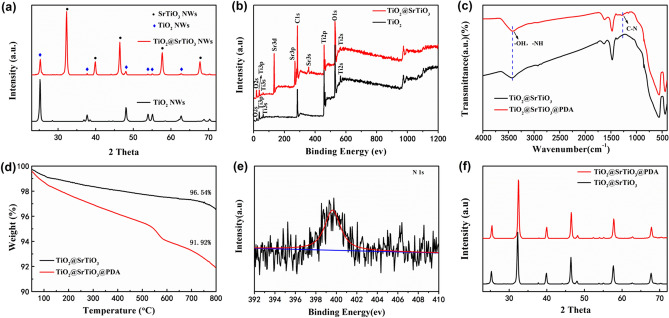
(**a**) XRD patterns and (**b**) XPS spectra of TiO_2_ NWs and TiO_2_@SrTiO_3_ NWs. (**c**) FT-IR spectra (**d**) TGA curves of TiO_2_@SrTiO_3_ NWs and TiO_2_@SrTiO_3_@PDA NWs. (**e**) High-resolution XPS spectrum of N_1s_ of TiO_2_@SrTiO_3_@PDA NWs. (**f**) XRD patterns of TiO_2_@SrTiO_3_ NWs and TiO_2_@SrTiO_3_@PDA NWs. This figure was created using OriginLab OriginPro 8.5 (https://www.originlab.com) and Microsoft Office PowerPoint 2007 (https://www.office.com).

The core–shell structured TiO_2_@SrTiO_3_ NWs were synthesized by TiO_2_ surface conversion. TEM and SEM images demonstrated that the SrTiO_3_ nanocubes were successfully encapsulated on the surface of TiO_2_ NWs, as shown in Fig. S1 and 2. Figure 2a,c show that the surface of pure TiO_2_ NWs is smooth. However, Fig. 2b as well as 2d shows that the smooth surface is uniformly covered by regularly-shaped SrTiO_3_ nanocubes after hydrothermal treatment in Sr(OH)_2_·8H_2_O solution. Based on SEM results, the average length and average diameter of TiO_2_/SrTiO_3_ NWs are calculated to be 6.3 µm and 320 nm, respectively (Fig. S2). As a consequence, the calculated aspect ratio of TiO_2_/SrTiO_3_ NWs approximates to 20. The large aspect ratio could decrease the percolation threshold of the NCs, achieving high energy density at a lower additive amount. The crystal phases of TiO_2_ NWs as well as TiO_2_@SrTiO_3_ NWs were analysed by the XRD patterns (Fig. 3a). The diffraction peaks of TiO_2_ NWs are consistent with the anatase TiO_2_ crystal structure according to the JCPDS card number 21–1272^31^. After hydrothermal treatment of TiO_2_ NWs, the products exhibit some additional peaks, which could be ascribed to the characteristic peaks of cubic SrTiO_3_ according to JCPDS card number 35–734^31^, indicating a successful TiO_2_ surface conversion. Besides, XPS spectra also indicated the generation of SrTiO_3_ on the surface of TiO_2_. XPS spectrum of TiO_2_@SrTiO_3_ showed peaks corresponding to Sr3d and Sr3p, which were not present in the XPS spectrum of pristine TiO_2_ NWs (Fig. 3b).

To better disperse TiO_2_@SrTiO_3_ NWs and make them more compatible with the PVDF matrix, dopamine was used as a surface modifier. The catechol and amino functional groups of dopamine can form covalent and non-covalent interactions with the surface of the TiO_2_@SrTiO_3_ NW, which lead to the dopamine adhere to the surface of the TiO_2_@SrTiO_3_ NW^32–34^. And oxidative self-polymerization of dopamine resulted in the formation of dense and robust layers on the TiO_2_@SrTiO_3_ NW surface (Fig. S3)^15,21,27,35^. The FT-IR and TGA analysis confirmed the successful coating of polydopamine on the surface of TiO_2_@SrTiO_3_ NWs. As shown in Fig. 3c, the FT-IR spectrum of TiO_2_@SrTiO_3_@PDA NWs shows a band that is absent in the FT-IR spectrum of TiO_2_@SrTiO_3_ NWs. The infrared absorption peak at 1268 cm^−1^ is attributed to the –C–N stretching vibration^27,36^. The signal belongs to dopamine and is not observed in the TiO_2_@SrTiO_3_ NWs. In addition, the peak at 3100–3700 cm^−1^, which is attributed to –NH and/or –OH stretching vibrations, becomes stronger in the TiO_2_@SrTiO_3_@PDA NWs compared with that in the TiO_2_@SrTiO_3_ NWs. These results indicate successful surface modification by dopamine. Due to the degradation of polydopamine which adheres to the TiO_2_@SrTiO_3_ NWs surface, the TiO_2_@SrTiO_3_@PDA NWs have a higher weight loss compared to the unmodified TiO_2_@SrTiO_3_ NWs (Fig. 3d). The surface elemental composition of the TiO_2_@SrTiO_3_@PDA NWs was further investigated by XPS analysis. Figure 3e shows the appearance of N_1s_ peak in the high-resolution XPS spectrum of TiO_2_@SrTiO_3_@PDA NWs, which confirms the presence of polydopamine on the TiO_2_@SrTiO_3_ surface^21^. The XRD analysis was used to investigate the crystal phases of the TiO_2_@SrTiO_3_ NWs and TiO_2_@SrTiO_3_@PDA NWs. As shown in Fig. 3f, the XRD pattern of TiO_2_@SrTiO_3_ NWs did not change after surface modification, indicating that the surface modification has no effect on the crystalline structure.

### Characterization of the TiO_2_@SrTiO_3_@PDA NWs/PVDF nanocomposites

**Figure 4:**
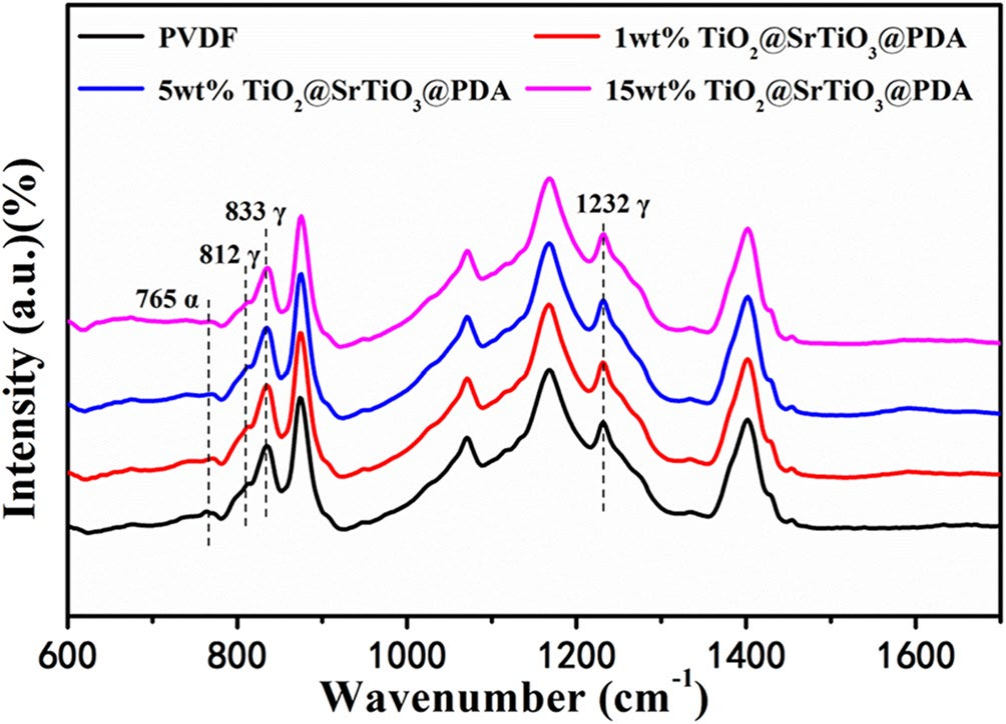
The FTIR spectra of PVDF and TiO_2_@SrTiO_3_@PDA NWs/PVDF NCs containing different contents of TiO_2_@SrTiO_3_@PDA NWs. This figure was created using OriginLab OriginPro 8.5 (https://www.originlab.com).

The FTIR spectra of TiO_2_@SrTiO_3_@PDA NWs/PVDF NCs containing different contents of TiO_2_@SrTiO_3_@PDA NWs are shown in Fig. 4. The FTIR spectra demonstrated that the crystalline phase of PVDF is mainly γ-phase. As seen from Fig. 4, all samples show strong infrared absorption peaks at 812, 833, and 1232 cm^−1^, indicating that γ-phase was formed in the samples. The peak at 765 cm^−1^ is ascribed to α-phase and remains unchanged after introduction of TiO_2_@SrTiO_3_@PDA NWs, indicating negligible phase transition from α-phase to γ-phase^37^. The polar γ-phase usually presents high breakdown strength, which is favorable for enhancing the energy density of the NCs^38^.

**Figure 5:**
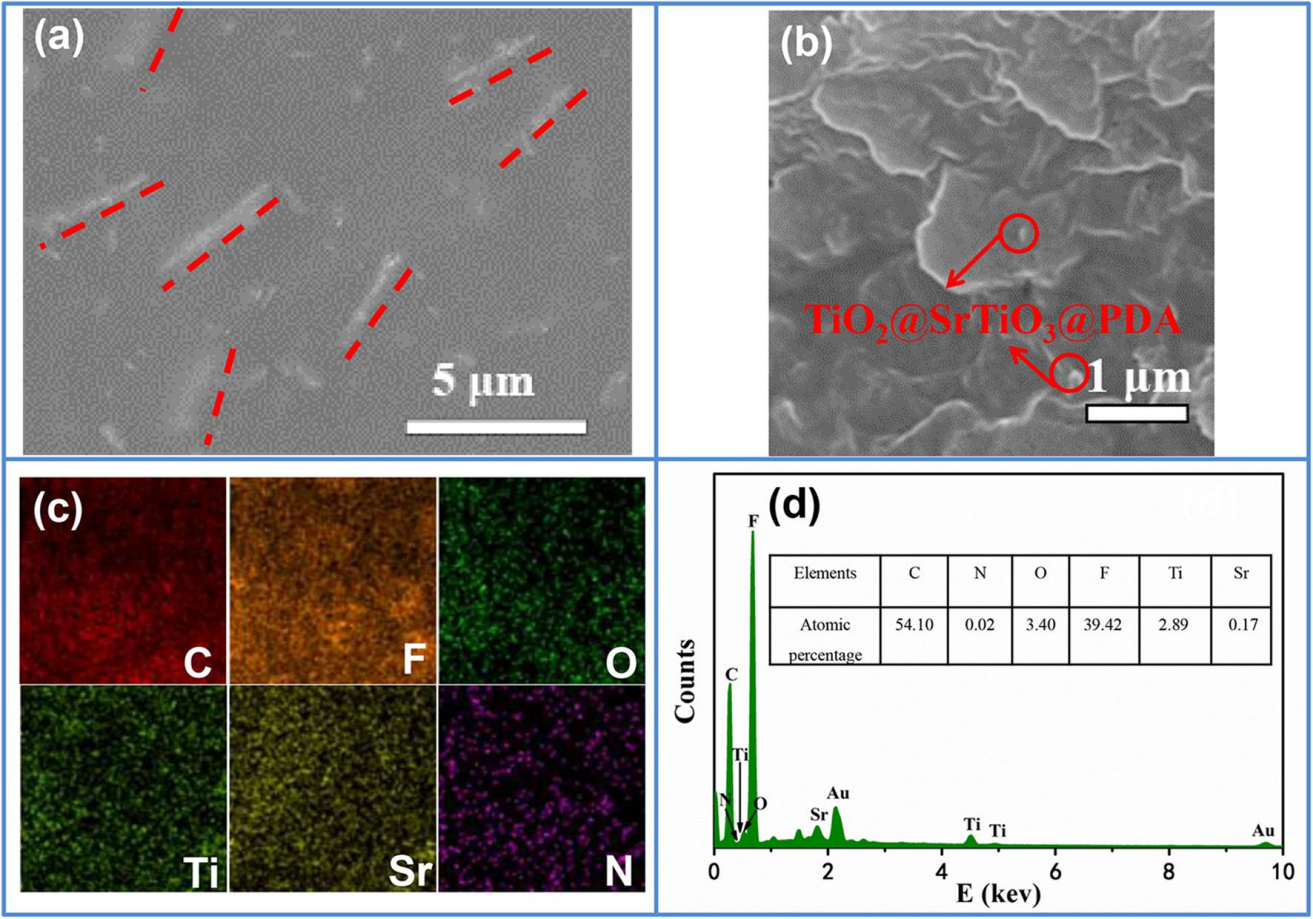
(**a**) The top-view and (**b**) cross-section SEM images of 5 wt% TiO_2_@SrTiO_3_@PDA NWs/PVDF NC. (**c**) The cross-section SEM mapping images of 5 wt% TiO_2_@SrTiO_3_@PDA NWs/PVDF NC. (d) EDS corresponding to the cross-section SEM mapping images. This figure was created using OriginLab OriginPro 8.5 (https://www.originlab.com) and Microsoft Office PowerPoint 2007 (https://www.office.com).

The SEM images and mapping images of the 5 wt% TiO_2_@SrTiO_3_@PDA NWs/PVDF NC are shown in Fig. 5. The SEM images show the nanofillers are dispersed homogeneously in the PVDF matrix with little agglomeration, and they orient in the in-plane direction relative to the PVDF matrix (Fig. 5a,b). This is beneficial for improving the breakdown strength and energy density of the NCs. Moreover, the film has a very small amount of defects (such as visible voids or flaws), which originates from the good interfacial compatibility between the PVDF matrix and nanofillers induced by hydrogen bonds between the PVDF and polydopamine. The cross-section SEM mapping images of the 5 wt% TiO_2_@SrTiO_3_@PDA NWs/PVDF NC further confirm that the distribution of nanofillers in PVDF matrix is homogeneous (Fig. 5c). The energy dispersive X-ray spectrum (EDS) corresponding to the cross-section SEM mapping images of 5 wt% TiO_2_@SrTiO_3_@PDA NWs/PVDF NC shows C, N, O, F, Ti and Sr peaks, as shown in Fig. 5d. Inset of Fig. 5d shows each elemental composition percentage.

### Thermal and crystallization behavior of the TiO_2_@SrTiO_3_@PDA NWs/PVDF nanocomposites

**Figure 6:**
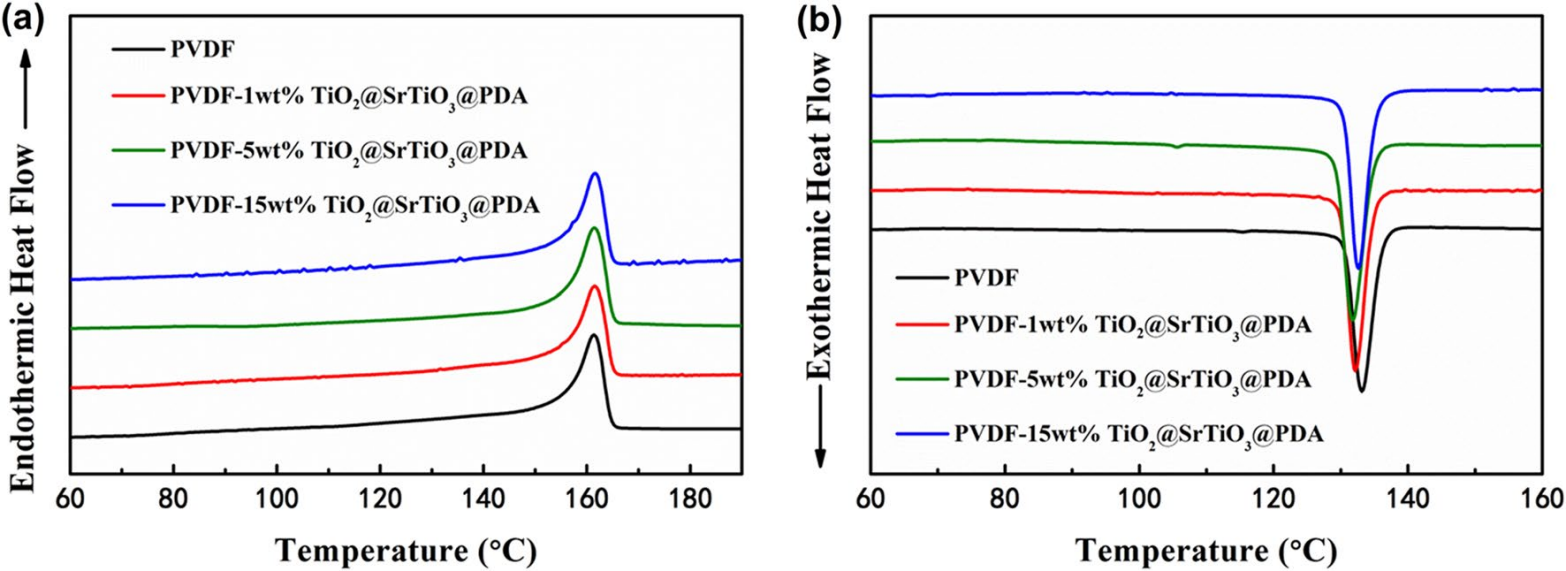
(**a**) Heating curves as well as (**b**) cooling curves of the pure PVDF and TiO_2_@SrTiO_3_@PDA NWs/PVDF NCs. This figure was created using OriginLab OriginPro 8.5 (https://www.originlab.com) and Microsoft Office PowerPoint 2007 (https://www.office.com).

DSC curves were used to analyze the influence of the weight fractions of TiO_2_@SrTiO_3_@PDA NWs on the crystallization behavior of the PVDF matrix. As shown in Fig. 6 and Table 1, the melting temperature and crystallization temperature of the NCs are slightly changed compared to the pure PVDF. The crystallinity (X_c_) of PVDF is calculated using the following Eq. ^39^:

3 $${\text{Xc }}\left( {\text{\% }} \right) = \frac{{\Delta {\text{H}}_{{\text{m}}} }}{{\left( {1 - {\text{w }}} \right)\Delta {\text{H}}_{{\text{m}}}^{{{ }0}} }} \times 100{\text{\% }}$$

**Table 1:** Crystallization temperature (T_c_), melting temperature (T_m_) and crystallinity (X_c_) derived from the DSC measurements of pure PVDF and TiO_2_@SrTiO_3_@PDA NWs/PVDF NCs in Fig. 6.

Samples	T_c_ (°C)	T_m_ (°C)	X_c_ (%)
PVDF	133.1	161.5	40.1
1 wt% TiO_2_@SrTiO_3_@PDA	132.1	161.5	46.5
5 wt% TiO_2_@SrTiO_3_@PDA	131.8	161.5	45.7
15 wt% TiO_2_@SrTiO_3_@PDA	132.6	161.5	43.1

where ∆H_m_ and ∆H_m_^0^ (equal to 104.7 J/g^40^) are melting enthalpies of the sample and a completely crystalline PVDF, respectively, and *w* is the weight percentage of the TiO_2_@SrTiO_3_@PDA NWs in the NCs. The crystallinities of polymer in the NCs are calculated and summarized, as shown in Table 1. The crystallinity is enhanced from 40.1% for the pure PVDF to 46.5% for the 1 wt% TiO_2_@SrTiO_3_@PDA NWs/PVDF NC. However, the crystallinity declines with further increasing the weight fractions of TiO_2_@SrTiO_3_@PDA NWs since nanofillers have a two-side influence on the crystallization behavior of the polymer matrix^39^. On one hand, the addition of TiO_2_@SrTiO_3_@PDA NWs provides more heterogeneous nucleation sites, thus reducing the nucleation energy and promoting the crystallization of the PVDF matrix. On the other hand, TiO_2_@SrTiO_3_@PDA NWs act as physical obstacles, hindering the PVDF polymer chain motions. All the NCs display a relatively higher crystallinity compared to the pure PVDF, which is attributed to the nucleation effect of TiO_2_@SrTiO_3_@PDA NWs as the main factor affecting the crystallization of PVDF matrix.

### Dielectric properties of the TiO_2_@SrTiO_3_@PDA NWs/PVDF nanocomposites

**Figure 7:**
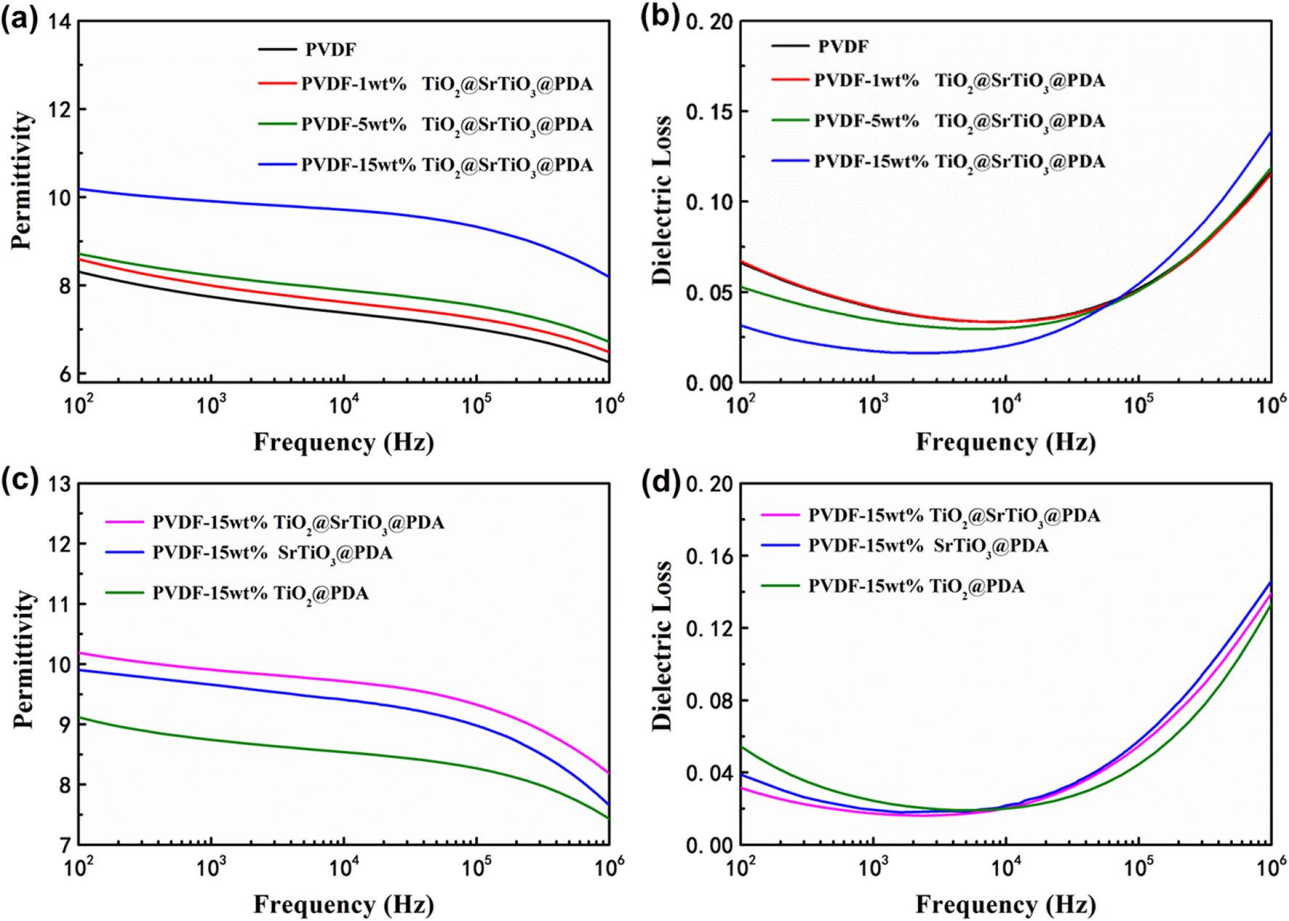
Frequency dependence of (**a**) permittivity and (**b**) dielectric loss of pristine PVDF and the TiO_2_@SrTiO_3_@PDA NWs/PVDF NCs. Frequency dependence of (**c**) permittivity and (**d**) dielectric loss of TiO_2_@SrTiO_3_@PDA NWs/PVDF, SrTiO_3_@PDA NWs/PVDF and TiO_2_@PDA NWs/PVDF with 15 wt% of fillers. This figure was created using OriginLab OriginPro 8.5 (https://www.originlab.com) and Microsoft Office PowerPoint 2007 (https://www.office.com).

**Figure 8:**
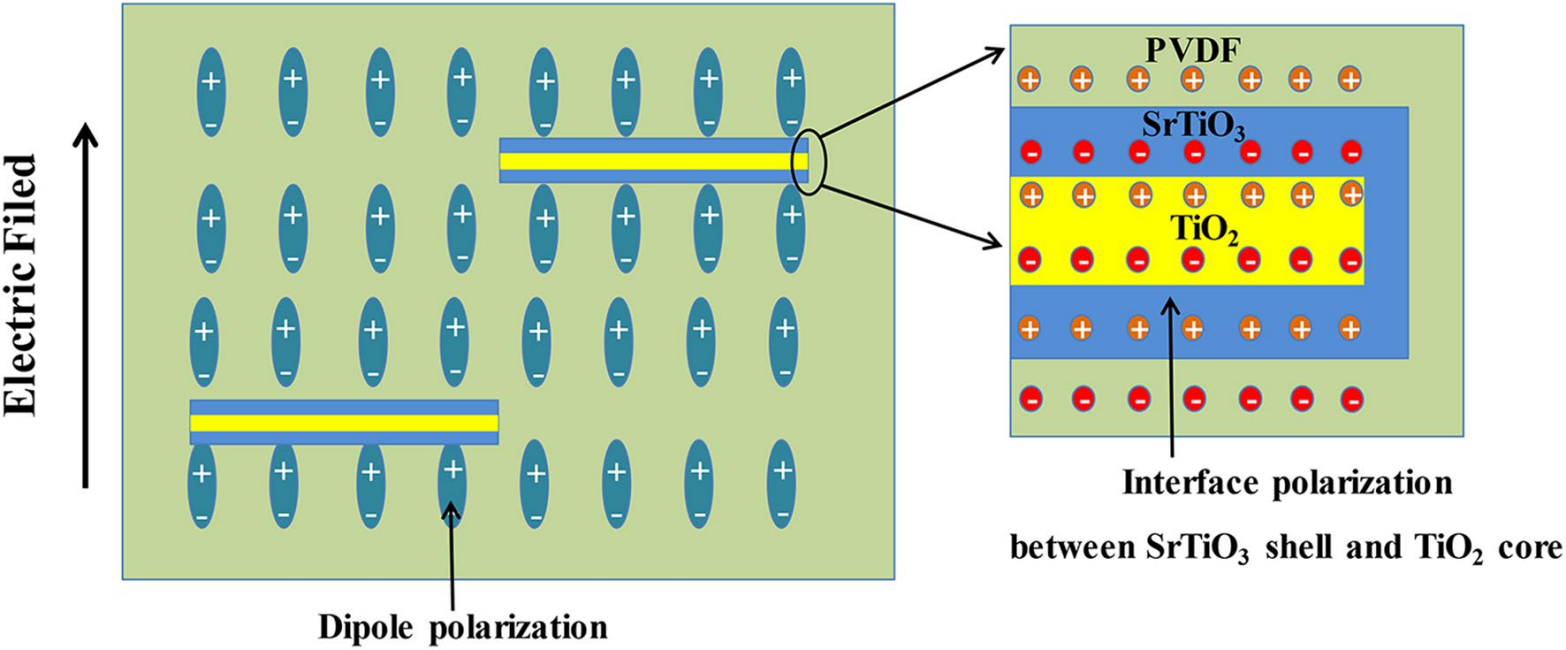
Dipole as well as interfacial polarization schematic for the TiO_2_@SrTiO_3_@PDA NWs/PVDF NCs under an external electric field. This figure was created using Microsoft Office PowerPoint 2007 (https://www.office.com).

The broadband dielectric spectrometer was used to measure the frequency-dependences of the permittivity and dielectric loss of PVDF and NC films with different weight fractions of TiO_2_@SrTiO_3_@PDA NWs. As shown in Fig. 7a, the permittivity of the NCs increases monotonously with increasing the content of nanowires, which can be interpreted as follows: (1) The permittivity of the large aspect ratio nanowires is higher than that of PVDF matrix. (2) The incorporation of TiO_2_@SrTiO_3_@PDA NWs into the PVDF matrix leads to hierarchical interfacial polarization in the TiO_2_/SrTiO_3_ interface and SrTiO_3_/PVDF interface, as shown in Fig. 8. In addition, with increasing the content of TiO_2_@SrTiO_3_@PDA NWs, the interfacial polarization increases, as a result, the permittivity in the NCs increases. For 15 wt% of TiO_2_@SrTiO_3_@PDA NWs/PVDF NC, the permittivity reaches up to 10.2 (at 100 Hz), which is larger than the value for pristine PVDF (i.e. 8.3 at 100 Hz). Meanwhile, the permittivity of the pure PVDF and NCs decreases with increasing the frequency since the dipoles of nanofillers and polymer cannot keep up with the change of external frequency as the applied electric field frequency increases^41^. As shown in Fig. 7b, due to decreasing the interface relaxation polarization loss, the dielectric loss of the NC films decreases as frequency increases in the 10^2^–10^4^ Hz range. However, in the 10^4^–10^6^ Hz range, the dielectric loss increases sharply as frequency increases due to the α_a_ relaxation related to the PVDF glass transition^42,43^.

To understand the effect of SrTiO_3_ shell on the dielectric properties of the NCs, the dielectric performances of the NCs with 15 wt% TiO_2_@SrTiO_3_@PDA NWs, SrTiO_3_@PDA NWs and TiO_2_@PDA NWs were studied. As shown in Fig. 7c, at the same frequency, the 15 wt% TiO_2_@SrTiO_3_@PDA/PVDF NC shows higher permittivity compared to the 15 wt% SrTiO_3_@PDA/PVDF NC and 15 wt% TiO_2_@PDA NWs/PVDF NC, which is ascribed to the additional interfacial polarization induced in the interfacial region of core–shell structured nanofillers. Due to the difference of the Fermi levels, permittivity as well as electrical conductivity between TiO_2_ and SrTiO_3_^44^, charge accumulates at the TiO_2_/SrTiO_3_ interface in the nanofillers when an electric field is applied (Fig. 8), causing Maxwell–Wagner–Sillars (MWS) interfacial polarization and the enhancement of the permittivity. Moreover, the 15 wt% TiO_2_@SrTiO_3_@PDA/PVDF NC has a lower dielectric loss than the 15 wt% SrTiO_3_@PDA/PVDF NC and 15 wt% TiO_2_@PDA NWs/PVDF NC at 100 Hz (Fig. 7d), which can be attributed to the influence of TiO_2_@SrTiO_3_ NWs core–shell structure.

### Breakdown strength of the TiO_2_@SrTiO_3_@PDA NWs/PVDF nanocomposites

**Figure 9:**
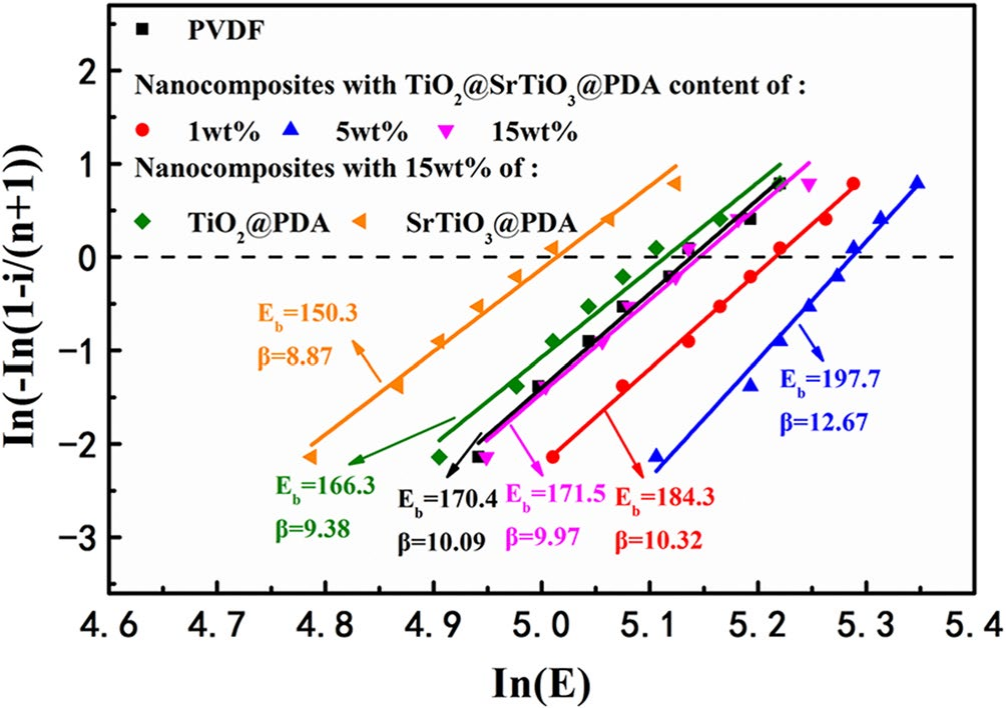
Breakdown strength Weibull plots of PVDF, NCs containing different contents of TiO_2_@SrTiO_3_@PDA NWs, 15 wt% SrTiO_3_@PDA NWs and 15 wt% TiO_2_@PDA NWs. This figure was created using OriginLab OriginPro 8.5 (https://www.originlab.com).

The breakdown strength plays an important role in determining the energy storage performance of dielectric materials. The breakdown strength of the PVDF and corresponding NCs is analyzed by Weibull statistics as follows^21,45,46^:

4 $${\text{P}}\left( {\text{E}} \right) = 1 - {\exp}\left[ { - \left( {\frac{{\text{E}}}{{{\text{E}}_{{\text{b}}} }}} \right)^{{\upbeta }} } \right]$$

where P(E) is the cumulative probability of electric failure, β quantifies the data scattering degree, E and E_b_ are experimental breakdown strength and characteristic breakdown strength (which is breakdown strength at the cumulative failure probability of 63.2%), respectively. Figure 9 shows breakdown strength Weibull plots of NCs containing different contents of TiO_2_@SrTiO_3_@PDA NWs, indicating that the introduction of TiO_2_@SrTiO_3_@PDA NWs in PVDF matrix can improve the breakdown strength of NCs. It can be observed that the highest breakdown strength of 198 MV/m can be achieved for the NC film containing 5 wt% TiO_2_@SrTiO_3_@PDA NWs, which is higher than the corresponding value for pure PVDF (170 MV/m). The enhanced breakdown strength of NCs can be interpreted as follows: (1) The large aspect ratio nanofillers orient in the in-plane directions of the PVDF matrix during solution casting, which might reduce the concentration of the electric field, act as ordered charge scattering centers and increase the tortuosity of the breakdown path^25,26^; (2) The SrTiO_3_ outer shell inhibits the adverse effects of TiO_2_ NWs on NCs, such as high electric conductivity, thus decreasing the leakage current density and dielectric loss; (3) Dopamine modification improves the dispersibility of the TiO_2_@SrTiO_3_ NWs as well as their compatibility with the PVDF matrix^15,21^. Besides, the breakdown strength of the NCs decreases as the weight fraction of nanofillers further increases, because the introduction of more nanofillers into the PVDF matrix inevitably results in more defects. To study the effect of SrTiO_3_ shell on the breakdown strength of the NCs, breakdown strength Weibull plot of the 15 wt% SrTiO_3_@PDA NWs/PVDF and TiO_2_@PDA NWs/PVDF are also shown in Fig. 9. The NC containing 15 wt% TiO_2_@SrTiO_3_@PDA NWs exhibits a higher breakdown strength than the NCs containing 15 wt% SrTiO_3_@PDA NWs and TiO_2_@PDA NWs, which is ascribed to the inhibition effect of SrTiO_3_ shell on TiO_2_ core. Most of the charges in the NCs containing TiO_2_@SrTiO_3_ NWs can only transfer in the interfacial region of core–shell structured nanofillers, leading to reduced electric percolation pathway and enhanced breakdown strength^3^.

### Energy storage performance of the TiO_2_@SrTiO_3_@PDA NWs/PVDF nanocomposites

**Figure 10:**
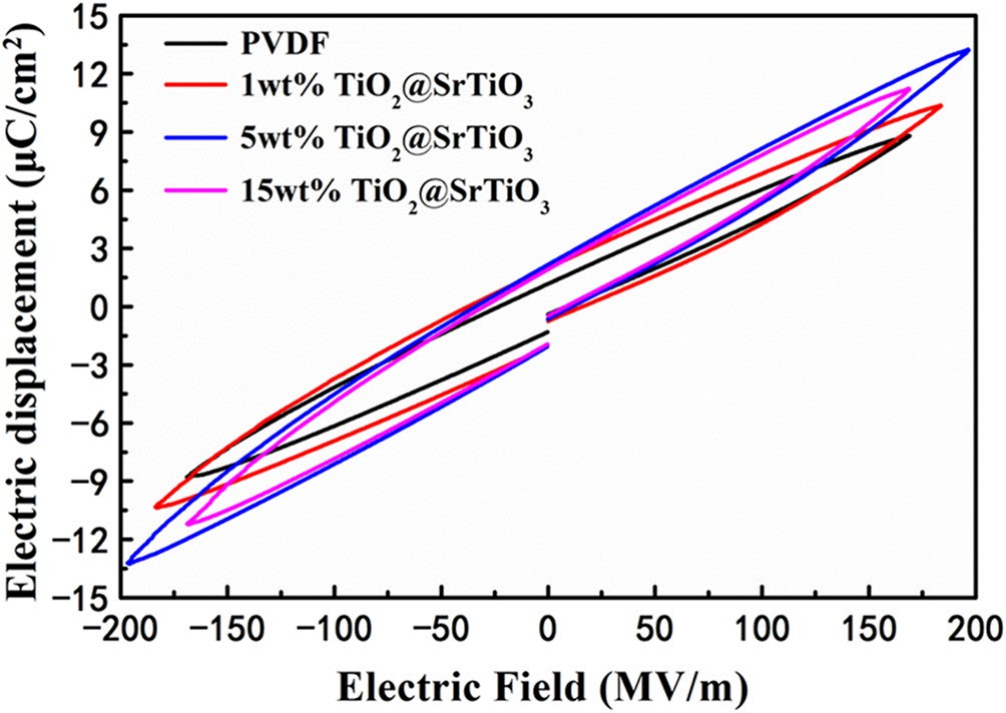
D–E loops of NCs filled with different weight fractions of TiO_2_@SrTiO_3_@PDA NWs at 100 Hz and room temperature before the NCs broke down. This figure was created using OriginLab OriginPro 8.5 (https://www.originlab.com).

**Figure 11:**
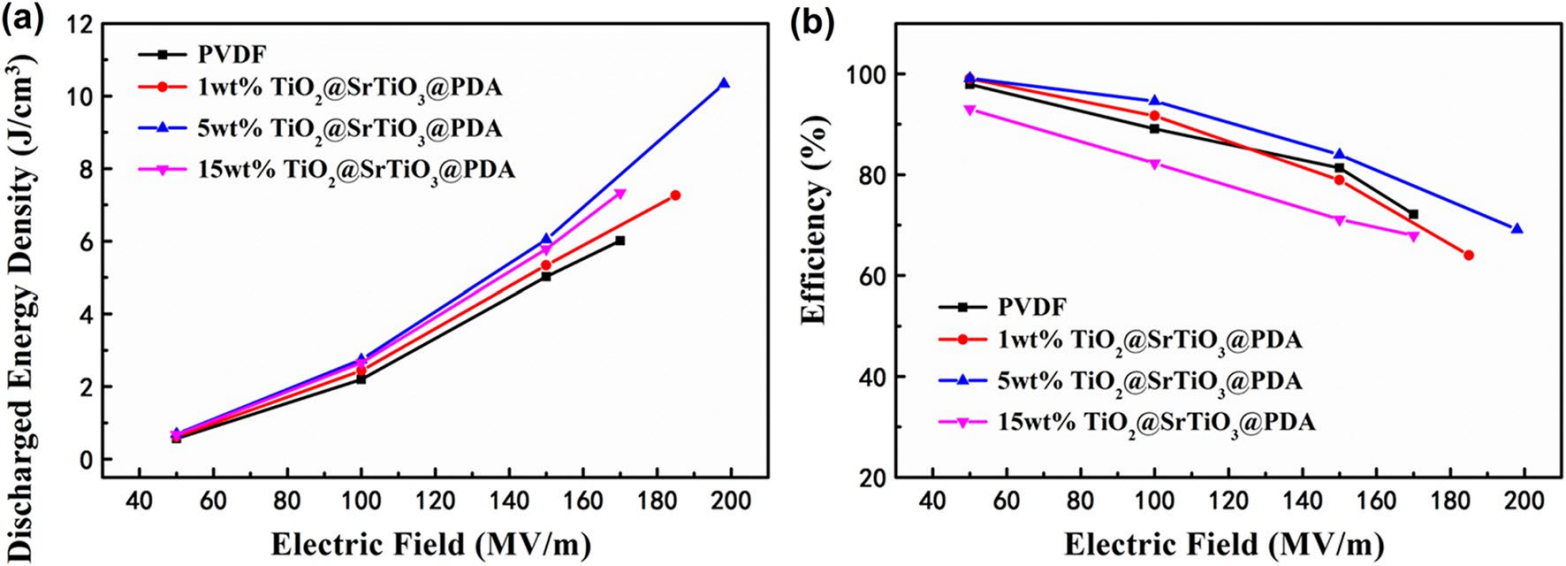
(**a**) Discharged energy densities and (**b**) charge–discharge efficiencies of PVDF-based NCs with different weight fractions of TiO_2_@SrTiO_3_@PDA NWs. This figure was created using OriginLab OriginPro 8.5 (https://www.originlab.com) and Microsoft Office PowerPoint 2007 (https://www.office.com).

To determine the energy storage performance, D–E loops of pure PVDF and the NCs are measured at 100 Hz as shown in Fig. 10 and S4. The introduction of the surface-modified TiO_2_@SrTiO_3_ NWs improves maximum electric displacement (Fig. S5a), due to the higher permittivity of TiO_2_@SrTiO_3_@PDA NWs and hierarchical interfacial polarization among TiO_2_, SrTiO_3_ and PVDF interfaces. The charged energy density and discharge energy density of the NCs are shown in Figs. S6 and 11a. Under the same electric field, the charged energy density of NCs increases with increasing the weight fractions of the nanofillers (Fig. S6). This can be ascribed to the high electric displacement of the NCs induced by the presence of the TiO_2_@SrTiO_3_@PDA NWs possessing high permittivity. It can be observed that the 5 wt% TiO_2_@SrTiO_3_@PDA NWs/PVDF NC exhibits maximum charged energy density of 14.95 J/cm^3^ at 198 MV/m, which is larger compared to that of pure PVDF (i.e. 8.34 J/cm^3^ at 170 MV/m) (Fig. S6). The maximum discharge energy density of 10.34 J/cm^3^ can be achieved in 5 wt% TiO_2_@SrTiO_3_@PDA NWs/PVDF NC at 198 MV/m, which is indeed 1.72 times larger than the corresponding value for pure PVDF (6.01 J/cm^3^ at 170 MV/m) (Fig. 11a). The largest discharge energy density of NC film originates from the simultaneous enhancement of the effective electric displacement (D_max_ − D_r_) and breakdown strength by the introduction of a small amount of dopamine-modified TiO_2_@SrTiO_3_ NWs (Fig. S5b and 9). Table 2 summarizes the energy storage performance of TiO_2_@SrTiO_3_@PDA NWs/PVDF NC and some previously reported dielectric NCs. It can be observed that the NCs in this study exhibit comparable or higher discharge energy density than that of previously reported dielectric NC films^16,47–57^. High discharge energy density is due to the additional interfacial polarization induced in the interfacial region of TiO_2_@SrTiO_3_ NWs and high permittivity and low remnant polarization of paraelectric ceramic SrTiO_3_ shell.

**Table 2:** Summary of the discharge energy density for various dielectric NCs containing different fillers.

Matrix	Fillers	Sample thickness (µm)	E_b_ (MV/m)	U_dis_ (J/cm^3^)	Refs.
PVDF	BaTiO_3_@Al_2_O_3_ nps	∼ 10	280	6.1	^47^
PVDF	BaTiO_3_@SiO_2_ nps	12–30	420	11.5	^48^
PVDF	TO@BT nps	10	380	8.78	^49^
PVDF	BaTiO_3_@Al_2_O_3_ nfs	10–15	380	7.1	^50^
PVDF	BT@AO-DA NFs	∼ 10	420	10.58	^51^
PVDF	BaTiO_3_@Al_2_O_3_ nfs	∼ 10	400	12.18	^52^
PVDF	TiO_2_–BT–TiO_2_@dopa	1−3	312.8	4.4	^53^
PVDF	BaTiO_3_/SiO_2_ nps	10–30	340	6.28	^54^
P(VDF-TrFE-CTFE)	TiO_2_@PZT	∼ 7	143	6.9	^16^
P(VDF-TrFE)	BaSrTiO_3_	30–40	155	4.72	^55^
P(VDF-HFP)	TO-450 NFs	∼ 50	160	7.63	^56^
PVDF	CCTO@Al_2_O_3_ NFs	∼ 15	340	8.46	^57^
PVDF	TiO_2_@SrTiO_3_@PDA	∼ 50	198	10.34	This work

Nanoparticles (nps); Nanofibres (nfs); BaTiO_3_@Al_2_O_3_-dopamine (BT@AO-DA); TiO_2_–BaTiO_3_–TiO_2_@dopamine (TiO_2_–BT–TiO_2_@dopa); TiO_2_@Pb(Zr_11x_Ti_x_)O_3_ (TiO_2_@PZT); CaCu_3_Ti_4_O_12_ (CCTO).

Both high discharge energy density and energy efficiency (η) of energy storage capacitors are desired for practical applications. The discharge energy efficiency (η) can be calculated by the following equation:

5 $${\upeta } = {\text{U}}_{{{\text{dis}}}} /{\text{U}}_{{{\text{stor}}}}$$

where U_dis_ and U_stor_ are the discharge and charge energy densities of the NCs, respectively. The discharge energy efficiencies of pure PVDF and the NCs are shown in Fig. 11b. The efficiency of the NC with 5 wt% surface-modified TiO_2_@SrTiO_3_ NWs is above 95% below 100 MV/m, and remains at 69% at 198 MV/m. Moreover, when the applied electric field increases, the efficiency of all NCs decreases due to the conduction loss.

**Figure 12:**
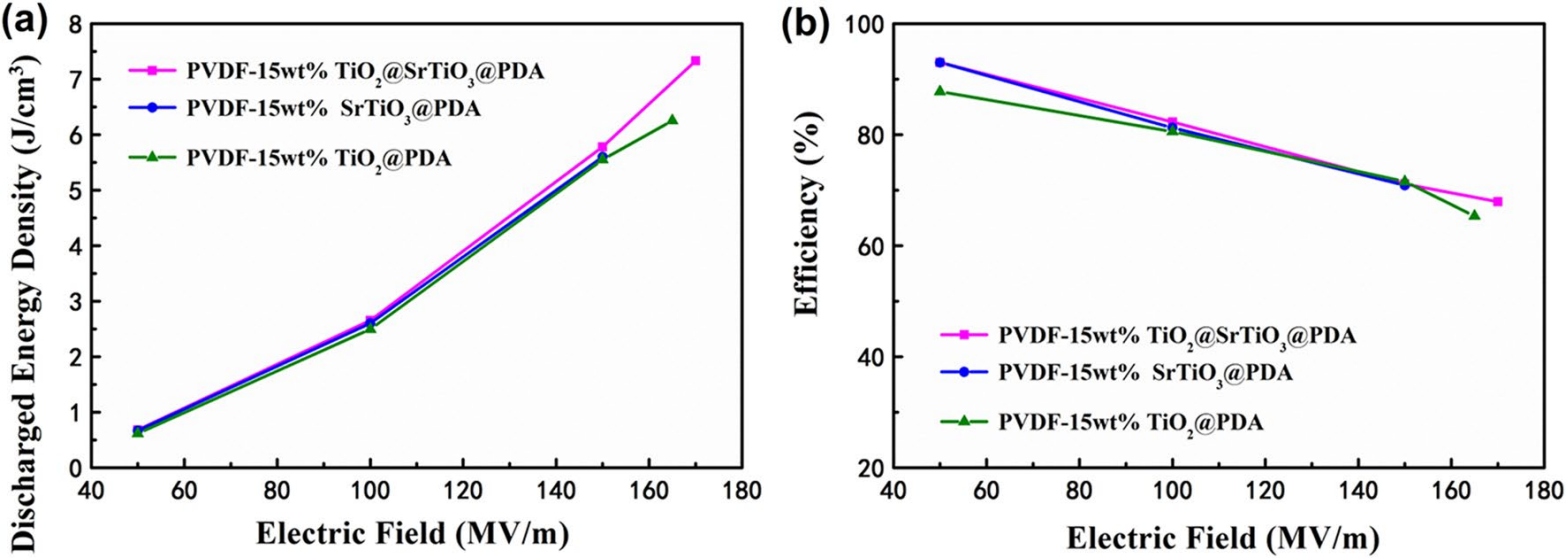
(**a**) Discharged energy densities and (**b**) charge–discharge efficiencies of TiO_2_@SrTiO_3_@PDA NWs/PVDF NC, SrTiO_3_@PDA NWs/PVDF NC and TiO_2_@PDA NWs/PVDF NC with 15 wt% of fillers. This figure was created using OriginLab OriginPro 8.5 (https://www.originlab.com) and Microsoft Office PowerPoint 2007 (https://www.office.com).

To understand the impact of SrTiO_3_ shell upon the energy storage capability, the energy density and charge–discharge efficiency of the NCs with 15 wt% TiO_2_@SrTiO_3_@PDA NWs, SrTiO_3_@PDA NWs and TiO_2_@PDA NWs were analyzed. The D–E loops of the 15 wt% TiO_2_@SrTiO_3_@PDA NWs/PVDF NC, 15 wt% SrTiO_3_@PDA NWs/PVDF NC and the 15 wt% TiO_2_@PDA NWs/PVDF NC were measured at 100 Hz as displayed in Figs. S4d and S7. Compared with the 15 wt% SrTiO_3_@PDA NWs/PVDF NC and 15 wt% TiO_2_@PDA NWs/PVDF NC, the 15 wt% TiO_2_@SrTiO_3_@PDA NWs/PVDF NC has a higher maximum electric displacement under the same electric fields (Fig. S8a), due to the additional interfacial polarization within the core–shell structure nanofillers. The charged and discharge energy density of the NC with 15 wt% TiO_2_@SrTiO_3_@PDA NWs are higher than those of NC with 15 wt% SrTiO_3_@PDA NWs and NC with 15 wt% TiO_2_@PDA NWs, as displayed in Fig. S9 and 12a. The NC with 15 wt% TiO_2_@SrTiO_3_@PDA NWs exhibits the superior discharge energy densities equal to 7.33 J/cm^3^ (at 170 MV/m), which is higher than discharge energy densities of the 15 wt% SrTiO_3_@PDA NWs/PVDF NC and 15 wt% TiO_2_@PDA NWs/PVDF NC (i.e. 5.60 J/cm^3^ at 150 MV/m and 6.25 J/cm^3^ at 165 MV/m, respectively). Compared to the 15 wt% SrTiO_3_@PDA NWs/PVDF NC and 15 wt% TiO_2_@PDA NWs/PVDF NC, the 15 wt% TiO_2_@SrTiO_3_@PDA NWs/PVDF NC has a higher effective electric displacement (D_max_-D_r_) and higher breakdown strength (Fig. S8b and 9), both of which contribute to the enhancement of the discharge energy density. Moreover, the 15 wt% TiO_2_@SrTiO_3_@PDA NWs/PVDF NC film has a higher charge–discharge efficiency than the 15 wt% SrTiO_3_@PDA NWs/PVDF NC film and 15 wt% TiO_2_@PDA NWs/PVDF NC film as shown in Fig. 12b. These results indicate that the core–shell structured TiO_2_@SrTiO_3_@PDA NWs are beneficial for the improvement of the energy storage performance of NCs.

### Mechanical properties of TiO_2_@SrTiO_3_@PDA NWs/PVDF nanocomposites

**Figure 13:**
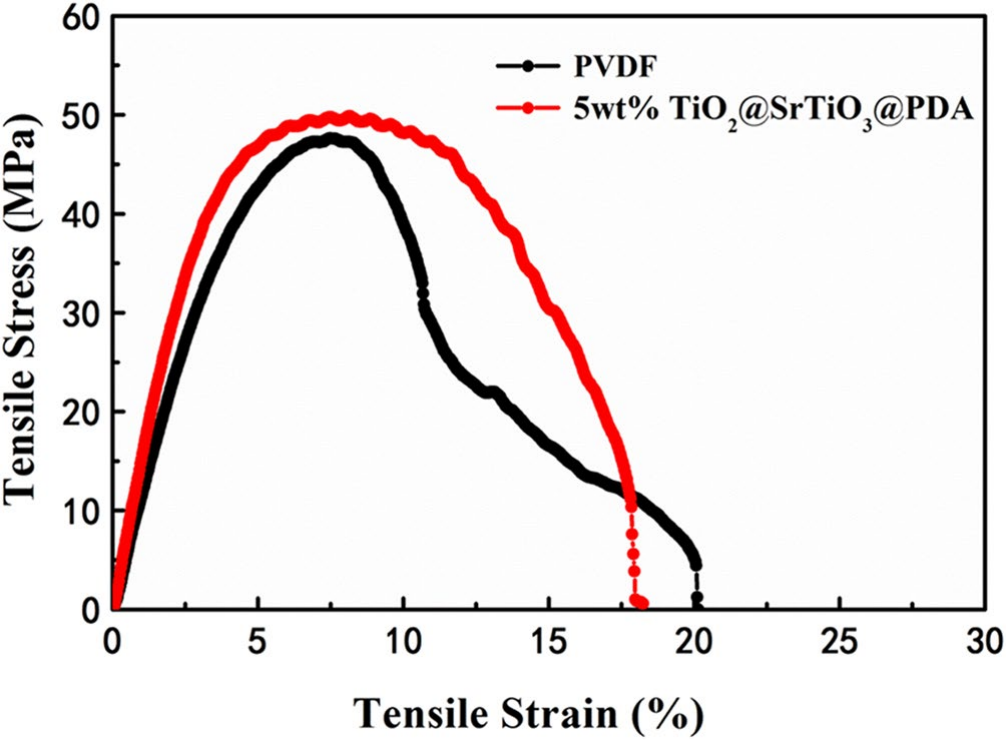
The stress–strain curves for the pristine PVDF and 5 wt% TiO_2_@SrTiO_3_@PDA NWs/PVDF NC at room temperature. This figure was created using OriginLab OriginPro 8.5 (https://www.originlab.com).

The mechanical properties of the NC films are an important parameter for practical applications. The mechanical properties of PVDF and 5 wt% TiO_2_@SrTiO_3_@PDA NWs/PVDF NC with excellent energy storage performance were investigated. Figure 13 shows the stress and strain curves of PVDF and the 5 wt% TiO_2_@SrTiO_3_@PDA NWs/PVDF NC. The elongation at break of the 5 wt% TiO_2_@SrTiO_3_@PDA NWs/PVDF NC is lower than that of pristine PVDF. There are two factors that explain this phenomenon. First, the TiO_2_@SrTiO_3_@PDA NWs can act as stress concentrators, providing the potential crack growth sites of the NCs. Second, the nanofillers can serve as physical obstacles that block the motion of polymer chains, leading to a brittle fracture^58^. Compared to pure PVDF, the 5 wt% TiO_2_@SrTiO_3_@PDA NWs/PVDF NC film has a larger tensile strength and tensile modulus due to the introduction of TiO_2_@SrTiO_3_@PDA NWs. As shown in Fig. 13, the tensile strength and tensile modulus of the NC with 5 wt% surface-modified TiO_2_@SrTiO_3_ NWs are 49.8 MPa and 1560 MPa, respectively, which are larger than those of pure PVDF (i.e. tensile strength of 47.6 MPa and tensile modulus of 1200 MPa). The researches show that the larger tensile modulus is, the higher the breakdown field is. Therefore, the increased tensile modulus is beneficial for the enhancement of discharged energy density in the NCs^59,60^.

## Conclusions

In this work, the NCs consisting of PVDF and functionalized TiO_2_@SrTiO_3_ NWs were fabricated by the solution casting technique. To improve the distributional homogeneity and compatibility between the nanofillers and PVDF matrix, the TiO_2_@SrTiO_3_ NWs were modified by dopamine. Thanks to the well-designed hierarchical interfacial polarization among their multiple interfaces, the large aspect ratio as well as surface modification of the TiO_2_@SrTiO_3_ NWs, the breakdown strength and electric displacement are simultaneously enhanced by incorporation of a small amount of TiO_2_@SrTiO_3_@PDA NWs, giving rise to high energy density of TiO_2_@SrTiO_3_@PDA/PVDF NCs. As a result, the maximum discharge energy density equal to 10.34 J/cm^3^ was achieved for the NC film containing 5 wt% TiO_2_@SrTiO_3_@PDA NWs at 198 MV/m, which is larger than the value for pure PVDF (i.e. 6.01 J/cm^3^ at 170 MV/m). Due to the introduction of TiO_2_@SrTiO_3_@PDA NWs, the tensile strength and modulus of the NC film are larger than those of pure PVDF. The results presented herein provide a good approach for the design the NC films with high energy storage capability and good mechanical properties.

## Supplementary information

Supplementary Information.
