# Supplementary material for: Enhanced energy density of PVDF-based nanocomposites via a core–shell strategy
Source: Sci Rep. 2020 Oct 13;10:17084. doi: 10.1038/s41598-020-73884-6 (PMC7555536; doi:10.1038/s41598-020-73884-6)
Supplement: Supplementary file 1 — Supplementary Information. [file 41598_2020_73884_MOESM1_ESM.docx]

**Supporting Information**

**Enhanced Energy Density of PVDF-based Nanocomposites via a Core-Shell Strategy**

Jingjing Xu,^ab^ Chao Fu,^ab^ Huiying Chu,^ab^ Xianyou Wu,^ab^ Zhongyang Tan,^ab^ Jing Qian,^ab^ Weiyan Li,^ab^ Zhongqian Song,^ab^ Xianghai Ran,^ab^* Wei Nie^ab^*

^a^ Lab of Polymer Composites Engineering, Changchun Institute of Applied Chemistry, Chinese Academy of Sciences, Changchun 130022, China

^b^ University of Science and Technology of China, Anhui 230026, China

*Corresponding authors.

E-mail address: [ranxh@ciac.ac.cn](mailto:ranxh@ciac.ac.cn) (X. Ran); [wnie@ciac.ac.cn](mailto:wnie@ciac.ac.cn) (W. Nie)


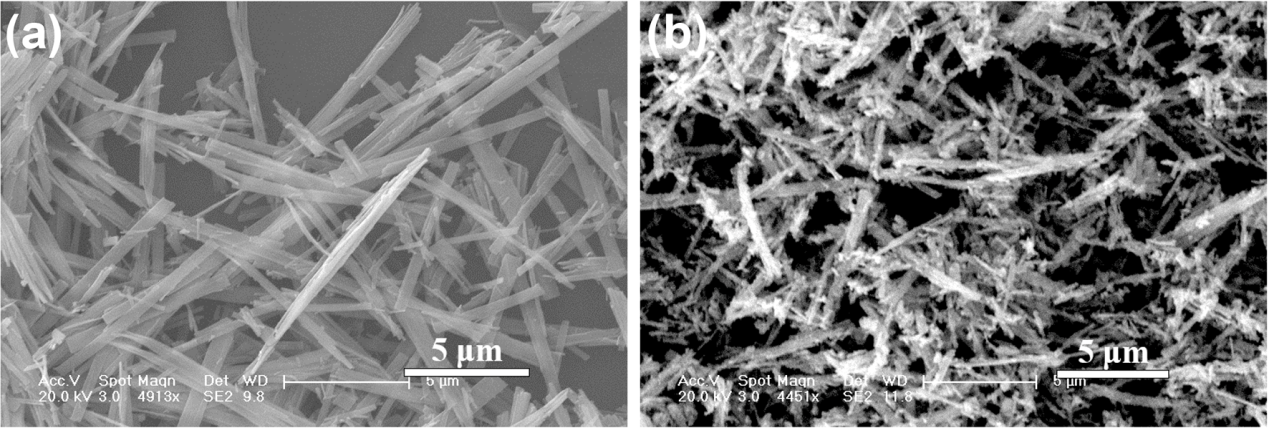


Figure S1. SEM images of (a) TiO_2_ nanowires and (b) TiO_2_@SrTiO_3_ nanowires.


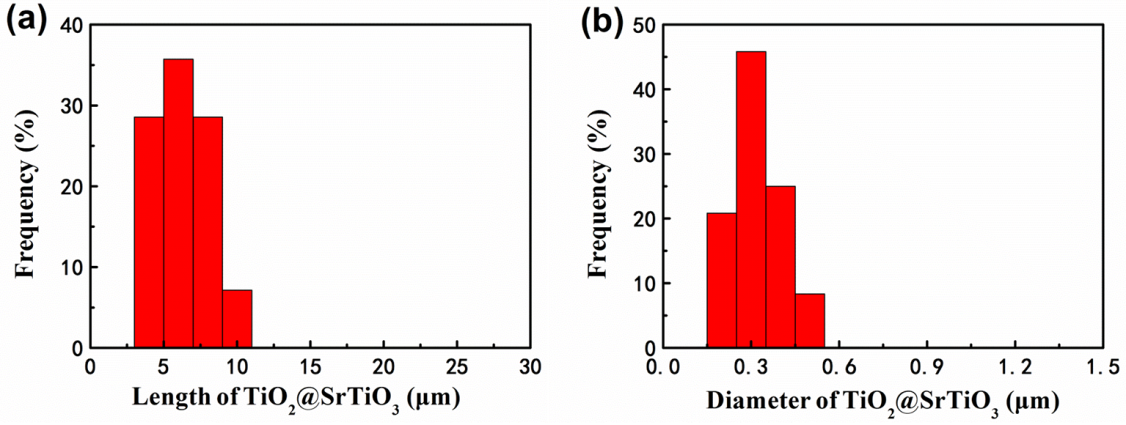


Figure S2 . (a) Length of TiO_2_/SrTiO_3_ NWs and (b) Diameter of TiO_2_/SrTiO_3_NWs.


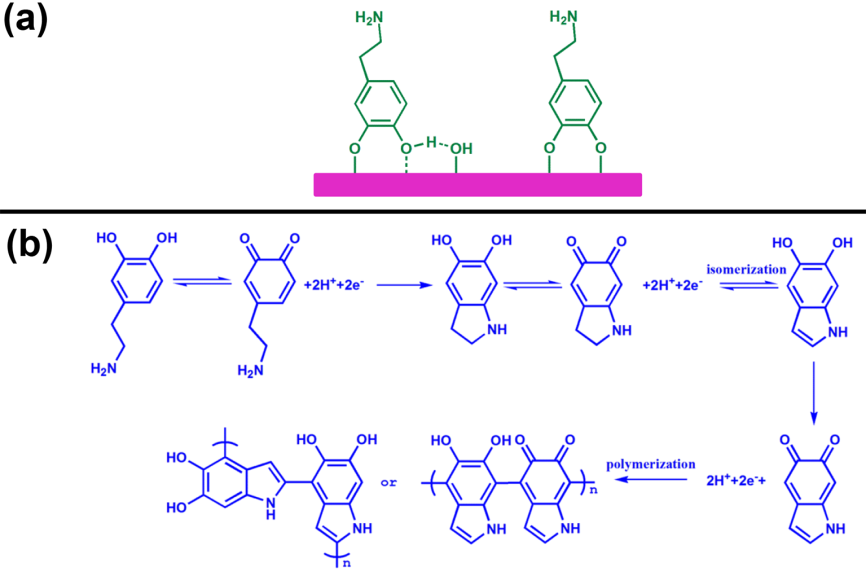


Figure S3. (a) Schematic of covalent and non-covalent interactions between dopamine and the TiO_2_@SrTiO_3_ NW surface (b) The mechanism for oxidative self-polymerization of dopamine.


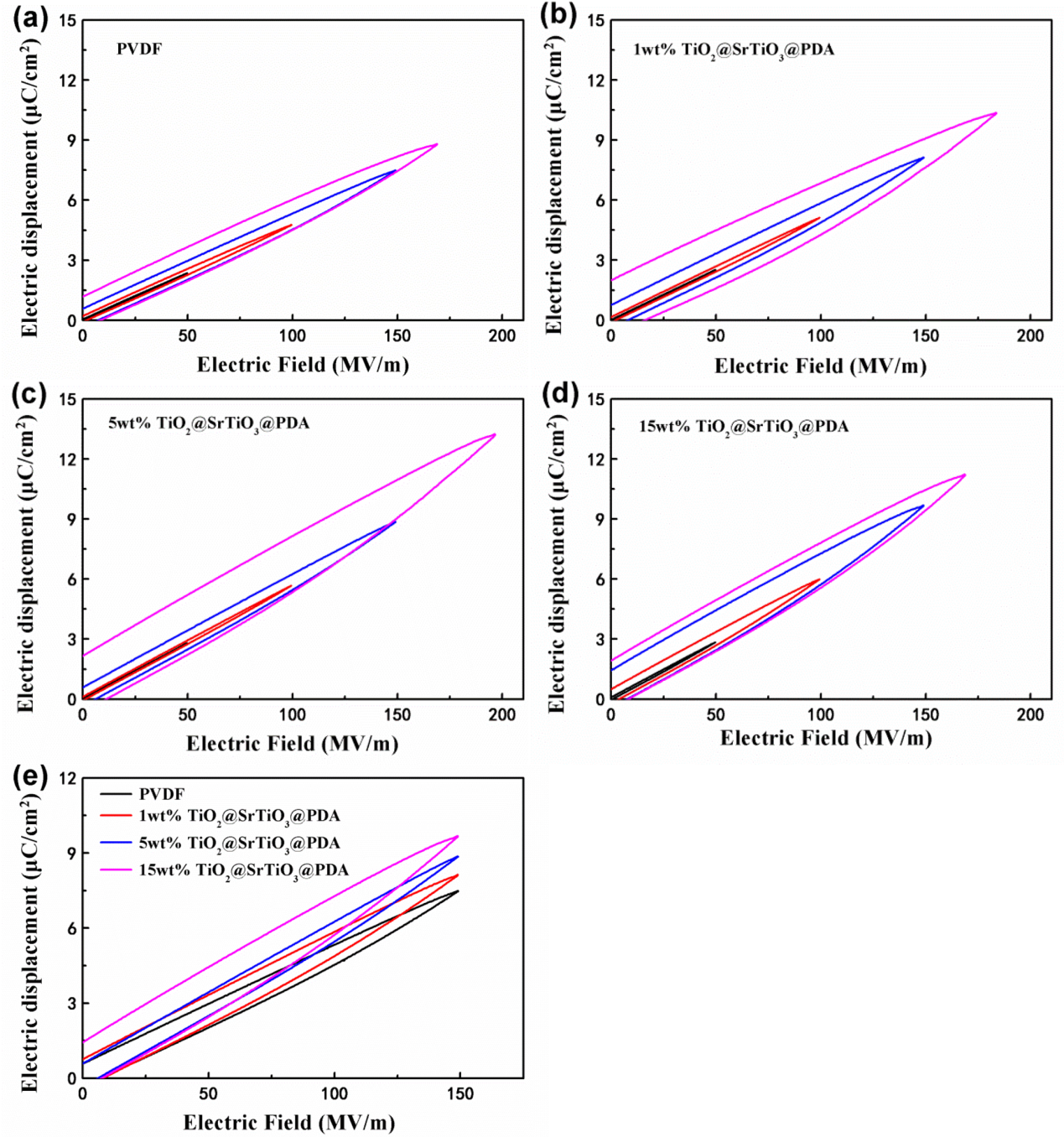


Figure S4. Electric displacement-electric field (D-E) loops at 100 Hz of (a) pristine PVDF, nanocomposites with TiO_2_@SrTiO_3_@PDA NWs content of (b) 1 wt%, (c) 5 wt%, and (d) 15 wt%. (e) Comparison of D-E loops of samples at an electric field of 150MV/m.


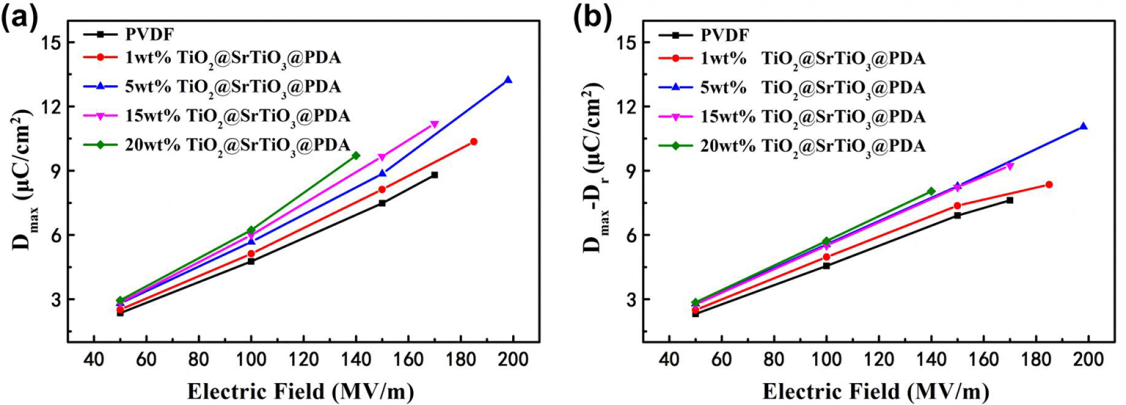


Figure S5. (a) D_max_ and (b) D_max_-D_r_ of PVDF-based nanocomposite films filled with different amounts of TiO_2_@SrTiO_3_@PDA NWs.


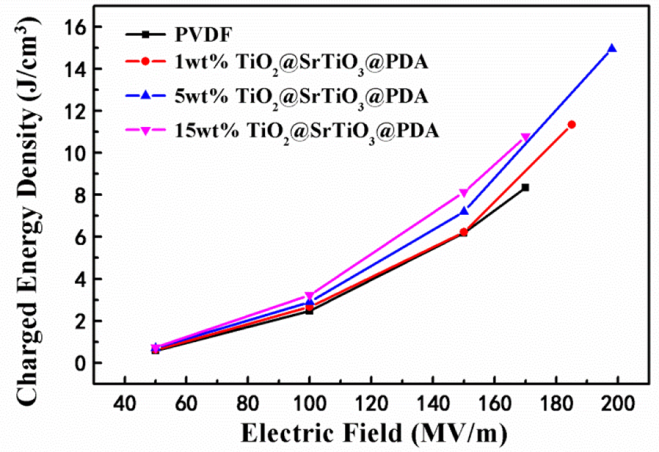


Figure S6. Charged energy densities of PVDF-based nanocomposites with different weight fractions of TiO_2_@SrTiO_3_@PDA NWs.


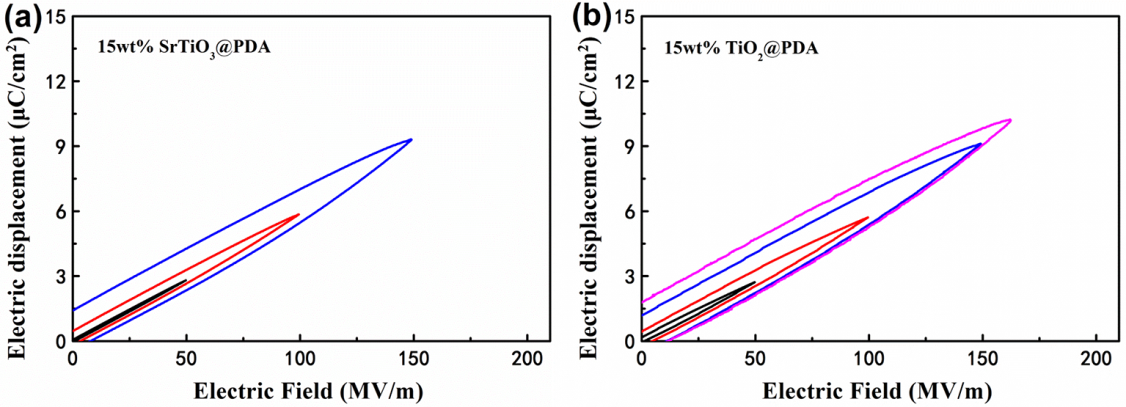


Figure S7. Electric displacement-electric field (D-E) loops of PVDF-based nanocomposite films loaded with (a) 15 wt % SrTiO_3_@PDA NWs and (b) 15 wt % TiO_2_@PDA NWs at 100 Hz.


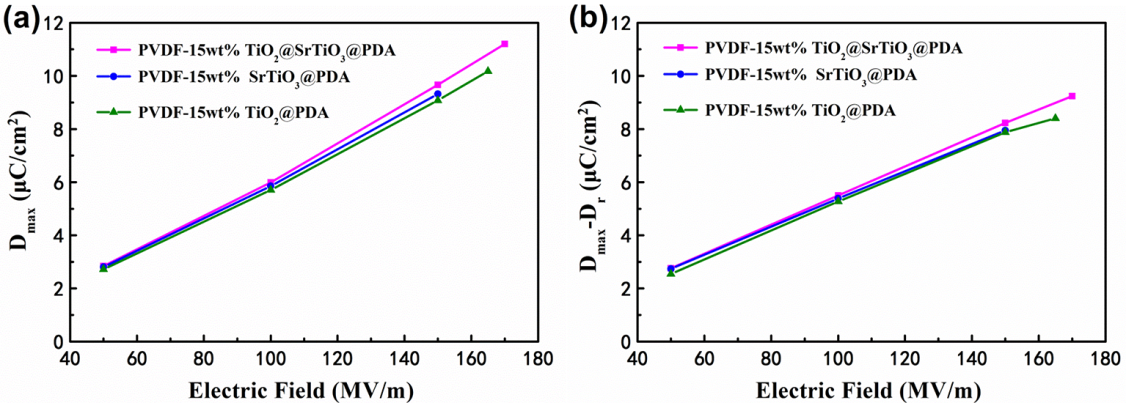


Figure S8. (a) D_max_ and (b) D_max_-D_r_ of PVDF-based nanocomposite films filled with 15 wt% TiO_2_@SrTiO_3_@PDA NWs, SrTiO_3_@PDA NWs and TiO_2_@PDA NWs.


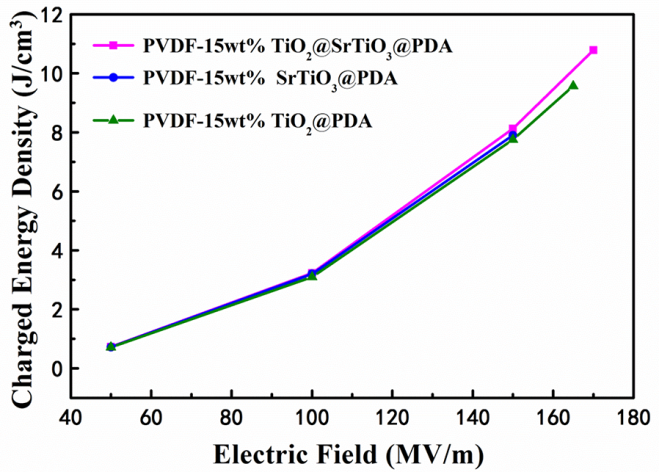


Figure S9. Charged energy densities of TiO_2_@SrTiO_3_@PDA/PVDF nanocomposite, SrTiO_3_@PDA/PVDF nanocomposite and TiO_2_@PDA/PVDF nanocomposite with 15wt% content of fillers.
